# The Many-Faced Program of Epithelial–Mesenchymal Transition: A System Biology-Based View

**DOI:** 10.3389/fonc.2017.00274

**Published:** 2017-11-13

**Authors:** De Domenico Stefania, Daniele Vergara

**Affiliations:** ^1^Biotecgen, Department of Biological and Environmental Sciences and Technologies, Lecce, Italy; ^2^Institute of Sciences of Food Production, National Research Council, Lecce, Italy; ^3^Department of Biological and Environmental Sciences and Technologies, University of Salento, Lecce, Italy

**Keywords:** epithelial–mesenchymal transition, cell plasticity, system biology, network analysis, regulatory networks

## Abstract

System biology uses a range of experimental and statistical methods to dissect complex processes that results from alterations in biological models. Given the complexity of the epithelial–mesenchymal transition (EMT) program, system biology represents a promising approach to understanding its fine molecular regulation by the interpretation of high-throughput datasets. Herein, we review recent contributions of system biology applied to the field of EMT physiology and illustrate the importance of these approaches to model biological networks that are perturbed during the transition. Together, these results allowed the definition of an EMT signature across different tumor types, the identification of dysregulated processes and new modules of regulation, making possible to reveal the EMT molecular visage underneath.

## Systems Approaches for Epithelial–Mesenchymal Transition (EMT) Complexity

**Figure 1:**
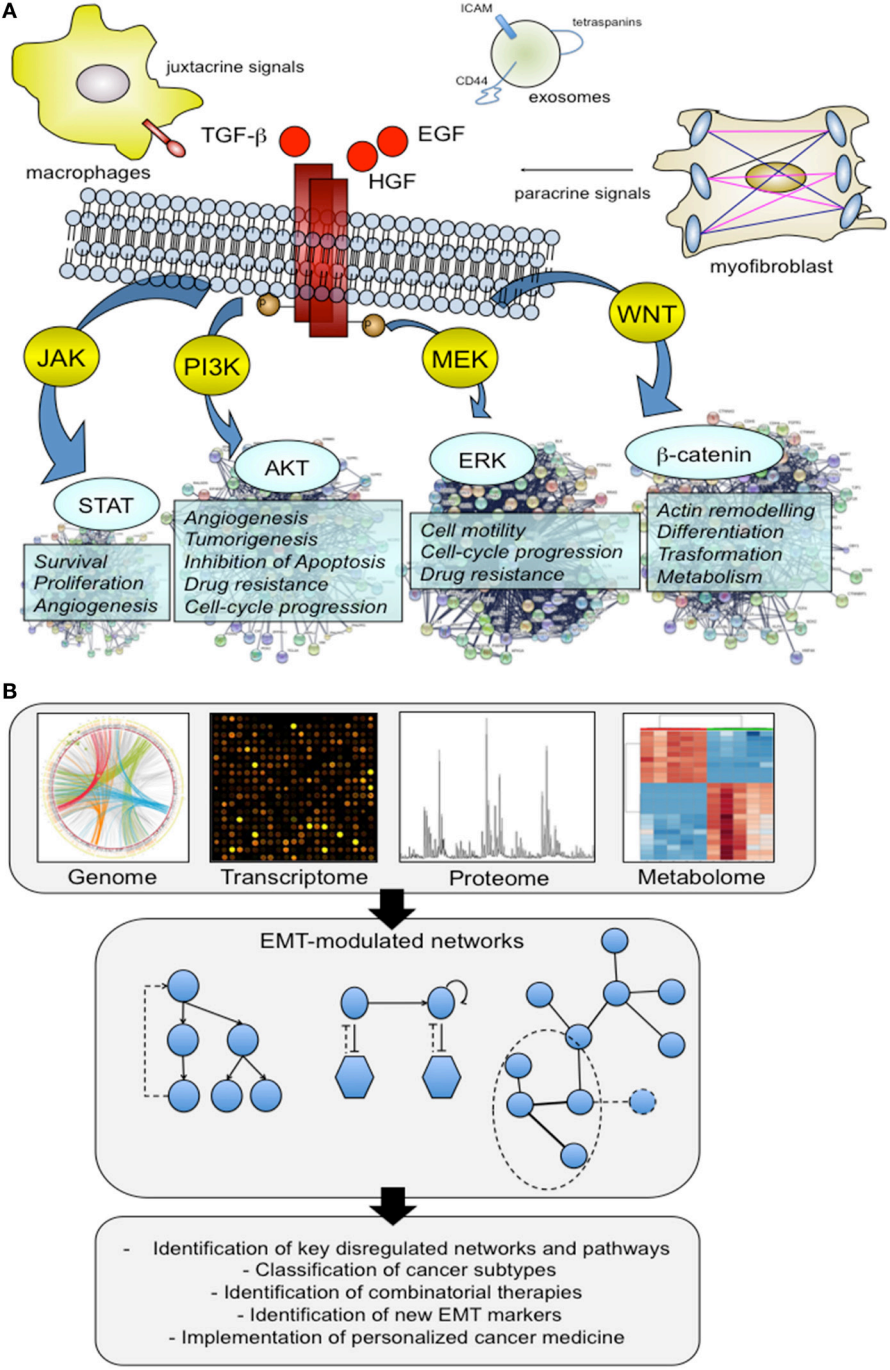
The multifactorial contribution of microenvironment to the activation of epithelial–mesenchymal transition (EMT). **(A)** We present below an illustrative but not comprehensive example of the different actors that may play a role in the activation of EMT process. Virtually, all the factors have the ability to direct a specific molecular program with a consequent activation of downstream signaling pathways capable of supporting and impacting multiple cell capabilities. Signaling proteins that include hepatocyte growth factor (HGF), epidermal growth factor, and transforming growth factor-β have well delineated, and an appreciated role. In addition, there is new evidence that other actors, such as tumor-associated macrophages (TAMs) and exosomes are able to promote the activation of the EMT program. TAMs act by creating a cancer stem cell niche through juxtacrine signaling, while exosomes function as cargo of molecules that drive cells toward an aggressive phenotype, more prone to EMT. **(B)** System biology approaches shed light on this complexity through the analysis of large patients’ cohorts and preclinical models at genomic and non-genomic level. The availability of high-throughput omics data has made possible the integration of different molecular datasets into functional networks that represent a tool to explore how nodes functionally interact with one another and to model perturbed networks generated from multilevel omics data. The obtained results made a substantial contribution to the definition of several aspects of EMT biology, including the identification of previously unknown EMT correlated pathways, and regulatory modules.

Normal cells show a structured organization that is finely regulated by a complex interplay between the systemic and local environment, and external factors. However, cells are also characterized by a certain grade of adaptation and plasticity to modify tissue organization. For example, under the control of a specific molecular program, endothelial cells activate different programs of differentiation (endothelial-to-hematopoietic cell transition and endothelial-to-mesenchymal transition) that play a major role during homeostasis but also during disease and different pathological stimuli (1). This phenotypic switching can easily be observed for other cell types including epithelial cells whose organization in normal tissues is guided by an epithelial polarity programme (EPP) (2). The execution of EPP is essential for the maintenance of epithelial polarization and differentiation status, and alterations of this program by EMT result in a cellular reprogramming with consequent fundamental alterations of cellular physiology. The main hallmarks of EMT can be summarized as follow: loss of cell–cell junctions, loss of cell polarization and acquisition of a front-rear polarity, and enhanced migratory capability. EMT morphological change reflects the actuation of a specific molecular program that is activated by juxtacrine or paracrine signals of the local environment that induce hierarchical, multilayered signaling networks both in physiological and pathological conditions (3) (Figure 1A). A number of mediators have been identified, including hormones, growth factors [for example, epidermal growth factor, and transforming growth factor-β (TGF-β)] as well as extracellular vesicles like exosomes that contain a unique molecular profile involved in the activation of EMT (4). These characteristics make EMT and the regulation by microenvironment extremely complex, resulting in a heterogeneous activation of several cellular pathways and networks (Figure 1A). This complexity is partially reduced if we consider that the EMT program is regulated at transcriptional levels through a defined set of transcriptional factors, but not completely solved because, as described later, this transcriptional program cannot be easily predict due to the presence of multiple layers and modules of regulation.

Thus far, three major types of EMT program have been described and classified according to the biological context in which they occur: type I associated with embryogenesis, type II to wound repair, and a type III to cancer pathology (5). The latter is by far the best-characterized type of EMT and the main topic of this review. EMT is indeed central to several aspects of tumor metastasis. For instance, insights into the biological complexity of EMT has been provided by genetic and biochemical studies, that associated this program to the different phases of the metastatic cascade, including tumor formation (6), early dissemination of tumor cells from the primary mass (7), and the acquisition of a chemoresistance phenotype (8, 9). EMT activation can also lead to the generation of stem cells in normal and cancer tissue (10, 11) through the activation of a specific transcriptional program (11). At the metastatic site, the cancer cell–stroma crosstalk drives mesenchymal cells to revert to an epithelial state through the activation of a mesenchymal-to-epithelial transition program thus allowing proliferation in distant sites (12, 13).

Since metastasis is the end result of a multistep process regulated through the activation of specific cellular and molecular programs, and as EMT has a role in most of these steps, this indicates that the contribution of EMT and the molecular aspects that govern EMT in these different phases might be more complicated than thought previously. Therefore, when we refer to EMT and to the plethora of cellular processes and biological functions that support the activation of the program, we should consider the contribution of EMT through these sequences of events that involves the generation of cancer stem cells, the dissemination of tumor cells, the metastatic colonization, and therapeutic resistance, especially in regard to biological similarities and differences that the EMT program exhibit. Moreover, the distinct genetic background of different tissues make further complicated *in vitro* and *in vivo* investigation of EMT.

Several solutions are now becoming available to face this complexity at systemic level. For example, computational approaches for predicting EMT transcriptional factor regulatory networks by mathematical modeling (14), data aggregation for the definition of an EMT expression signature (15–19), integrative data analysis to collect and organize high-throughput experimental data and metadata (20, 21), networks and pathways analysis to explore high-complex data and to reduce data complexity. This latter approach has been applied to different cancer datasets to study genes/proteins altered after EMT markers knockdown (22, 23), to identify subtype-specific driver networks across different cancer subtypes (24), and between primary tumors and circulating tumor cells (25), to propose regulatory hubs with a potential impact on patient management (26, 27). This experimental strategy includes the identification of networks of pathways that are enriched in a dataset of differentially expressed genes, and/or the construction of interaction networks to map genes and proteins interactions (Figure 1B). To do this, multiple omics data, derived from *in vitro* and *in vivo* studies, were analyzed and integrated. Human cancer cell lines characterized for the expression of EMT markers are frequently used in EMT studies to identify a list of candidate networks or sub-networks to prioritize (28, 29). These results provided a systemic investigation of a large repertoire of cellular functions and processes modulated during EMT that go beyond activation of classical pathways of proliferation and survival, cell adhesion, and motility but that include novel network modules with a potential functional significance. Other studies used cancer cell lines to describe by mathematical models the different EMT phenotypic forms. This analysis reinforces the hypothesis that EMT is a multistep dynamic process that occurs through five different dynamically interconnected states (30). The transition between these different phenotypes is not casual, but finely regulated by specific regulatory circuits including the miR-34/Snail and the miR-200/Zeb modules (31). The proposed computational method was also applied to integrate different regulation modules. By integrating networks for different migratory phenotypes, it is possible to predict the switch from a mesenchymal type to the amoeboid one. This model integrates the EMT core module, through the miR-200 and miR-34, with the RhoA/Rac1 circuit (the core circuit for the amoeboid-to-mesenchymal transition and mesenchymal-to-amoeboid transition) to model and predict the transition to a different migration state (32).

However, this experimental approach is limited by the multicellular complexity of EMT that cannot be completely addressed by *in vitro* 2D assays that fail to adequately represent the structural architecture of complex tissues. Consequently, a reliable systemic representation of tumor–stroma interaction was obtained by pathways and networks analysis of *in vivo* models (33). In colorectal cancer, tumor buds and their surrounding stroma were isolated using laser microdissection and analyzed to identify an integrated TGFβ/Snail and TNFα/NFκB pathway that regulates the crosstalk between cancer cells and the microenvironment during the EMT process.

At the tissue level, genomic cancer data from The Cancer Genome Atlas (TCGA) represented also an important resource for the identification of cancer subtypes with epithelial and mesenchymal features, where a robust EMT gene expression signature was identified (34). When integrated with proteomics data, proteogenomics provided substantial improvements in the definition of coexpression networks (35). This combined approach led to a clear identification of an EMT profile across different tumor types, and further demonstrated how this signature was associated with a poor prognosis (36).

A comprehensive investigation of TCGA data was also performed to identify metabolic features of tumor samples. A gene set enrichment analysis was applied to a curated metabolic database to identify cancer-relevant metabolic pathways and to correlate metabolic alterations with the clinical outcome of cancer patients (37). This study demonstrated that loss of oxidative phosphorylation-related genes correlated significantly with EMT, and with a concomitant downregulation of specific mitochondria metabolites.

## System Approaches for Addressing EMT Complexity at Transcriptional Level: The EMT Face Changing Program

The recognition of EMT in tissues relies on the detection of specific epithelial and mesenchymal markers including cell-surface markers such as E-cadherin and cytoskeletal markers such as Vimentin and α-smooth muscle actin (5). However, this strict distinction relies on the hypothesis that cells that have undergone EMT have acquired a full mesenchymal state. While this is likely to be characteristic of specific tumor types that show molecular hallmarks of an active EMT program, i.e., triple-negative breast cancer tumors, this is not generally observed *in vivo* where a greater plasticity with different EMT states of transition is observed (38). Thus, rather than considering the expression of few biomarkers, these observations led to the definition of an EMT gene signature whose expression was significantly associated *in vivo* and *in vitro* with different EMT states and validated in different tumor types (15, 16), or predictive of response to EGFR and PI3K/Akt inhibitors (17), or potentially useful for the selection of patients who may benefit from immune checkpoint blockade (18). This approach not only identified a pan-EMT signature but also provided an experimental and functional validation of selected EMT markers (19). The most important challenge is how to decipher the molecular mechanisms behind the expression of these markers to clarify networks and pathways modified during EMT. There is now evidence that the expression of EMT biomarkers is temporally and spatially coordinated through the activation of a network of transcriptional factors (EMT-TFs) including Prxx1, Snail, Slug (Snail2), Twist, Zeb1, and Zeb2 that initiates and orchestrates the EMT program (39). There are several challenges in reconstructing and analyzing an EMT regulatory network. One major problem is that although some TFs can be coexpressed, the relationship between some of these might be unclear. The precise role of these TFs can be difficult to characterize as experimental data obtained in several works described for each of them specific functions that are not redundant but complementary and supported a regulatory hierarchy that is required for the initiation of EMT *in vitro* and *in vivo* (40). For example, the EMT–TFs expression profile of different tissues may be widely different, and the activation of a single and specific TF may be crucial to support EMT activation (41). Moreover, stimulation from the microenvironment or specific oncogenic factors could switch on the expression of a particular TF (42, 43). Activation of the NRAS/BRAF signaling pathway in melanoma cells drives a transition from Zeb2 and Snail2 proteins, which are unrelated to the disease, toward a Zeb1 and Twist high-expression pattern that is determinant for the oncogenic transformation and correlated with the level of malignancy (43). Oncogenic mutations can also affect the TFs activity in some cancers. For example, mutations in genes encoding for KRAS of EGFR oppositely regulate the activity of Zeb1 through molecular mechanisms that are independent of EMT (44). Therefore, to reveal the potential role of each TF and to translate this in the clinical setting, the cellular context and the local microenvironment should be considered. Finally, additional regulation of EMT–TFs activity is achieved through various posttranslational modifications (45), or through the binding with specific transcriptional activators such as the Hippo factor effector YAP (46).

Epithelial–mesenchymal transition–TFs are involved in the control of multiple biological activities and signaling networks in addition to the well-known role of regulators of cell junction-associated genes including E-cadherin. The current availability of high-throughput genomics and proteomics techniques makes it possible to uncover novel TFs that were not previously identified or to reveal biological functions affected by TFs expression. *In silico* comparative analysis of gene expression data of 762 cancer cell lines had a significant impact on the identification of novel EMT–TFs. In this analysis, a set of 25 EMT-dependent regulators of E-cadherin has emerged with relevance for the definition of a pattern of EMT–TFs across the entire data cohort. Importantly, not only this analysis identified a core EMT–TFs signature but it also provided an experimental validation of *in silico* predictions. Specifically, these findings demonstrated that Krüppel-like factor 5 is a novel candidate regulator of EMT that maintains epithelial characteristics by regulating E-cadherin expression (47).

In addition to such large dataset analysis, more recent system methods focused on the analysis of selected TFs. Approaches to addressing this issue include the overexpression of specific TFs in appropriate cellular models together with high-throughput strategies for the detection and quantification of modulated networks. This strategy was successfully used for the analysis of Snail that was overexpressed in a breast cancer cell model. This work involved the analysis by LC-MS/MS of proteins isolated and fractionated from MCF-7 cells (48). Protein networks analysis of differentially expressed proteins identified three main cellular processes modified after Snail induction, including energy metabolism, cell cycle, and chromatin binding and remodeling. From this network, authors identified two specific hubs, CDK1 and HDAC1 that are functionally associated with Snail (48). Another approach applied a computational modeling method to define the migration characteristics of human mammary epithelial cells before and after Twist induction (49). Twist induces an EMT-dependent migratory phenotype in response to the stimulation with a panel of growth factors that can be predicted *a priori* by a mathematical model that correlates the intracellular signaling activities to the phenotypic response (49).

These network-oriented experimental approaches are remarkable examples of a strategy aimed at reducing EMT complexity to a defined set of altered processes with several advantages, among these the possibility to interpret and compare core regulatory networks across different tumor types, as demonstrated for the EMT–TF E2F1. In bladder and breast cancer, a network approach distinguished two different receptor protein signatures associated with the E2F1-mediated EMT, E2F1–TGFBR1–FGFR1 in bladder cancer and E2F1–TGFBR2–EGFR in breast cancer. This *in silico* prediction was validated functionally *in vitro* by an shRNA-based approach, and *in vivo* by two TCGA cancer cohorts (50). Importantly, this analysis provided relevant aspects about the EMT–TFs regulation across different cancer types with important implications on tumor type-specific treatments.

Tumor microenvironment drives and modulates the activity of EMT–TFs and downstream target genes. TGF-β is a well-known EMT inducer acting through a network of signaling pathways. To explain the mechanisms of TGF-β-induced EMT, several studies integrated experimental and computational approaches to model the transition between the epithelial and mesenchymal state after TGF-β stimulation (51). For example, the CBS model consisting of “cascading bistable switches” operates through multiple feedback loops that involve the action of Snail, and Zeb1 and the regulation by two microRNA, miR-34 and miR-200 (51). The Snail/miR-34 feedback loop initiates the EMT transition, while Zeb1/miR-200 loop controls the establishment of a full mesenchymal state. More recently, this model was further implemented by considering the role of other TFs. For example, the mutual inhibition relationship between Ovol2 and Zeb1 was statistically described and experimentally validated (52). However, this model does not enable to explain completely the mechanisms underlying changes between different EMT phases, and other TFs should be considered to provide new insights into these biological differences. A computational analysis that integrated time-course transcriptomic data with public cistromic data enabled the identification of key TGF-β EMT regulators and correlated their expression to the acquisition of a partial and full mesenchymal state (53). ETS2, HNF4A, and JUNB autoregulate and positively regulate each other and are part of the same transcriptional complex, as experimentally validated (53). The construction of an accurate model of TGF-β-mediated gene regulation, took also in account modulated signaling networks. TGF-β signals through a pathway that involves SMAD proteins, and other non-canonical pathways, that are activated upon ligand–receptor activation. In the context of TGF-β EMT activation, the relative importance of these pathways was predicted through a network simulation. Importantly, the contribution of two canonical pathways, Wnt and sonic hedgehog that mediate EMT in a SMAD-independent way, was demonstrated (54). This has important implications in the therapeutic approach of EMT, to simplify the design of drug combinations that target multiple nodes of regulation. Importantly, the results of *in silico* analysis and siRNA validation demonstrated that a combinatorial strategy provided a synergistic effect at preventing TGF-β EMT activation, in contrast to the individual node perturbation of SMAD complex alone (26).

The interaction between tumor cells and the associated stroma regulates EMT initiation and progression, through the action of different stimuli (Figure 1A). One challenge when studying this interaction is to evaluate at system level these global changes. Mathematical models can be applied to manage this complexity and to predict how different factors may alter the EMT response. In detail, such approaches provided information about the dynamics of the networks modulated after the stimulation with TGF-β and/or vascular endothelial growth factor A. Cells acquired a mesenchymal or epithelial phenotype when stimulated with single agents, whereas exhibited a hybrid phenotype when costimulated. This transition phase was assumed to depend on the activity of the transcriptional factors Sp1 and NFATc, which coordinate the expression of epithelial and mesenchymal markers (55).

## Conclusion

Epithelial–mesenchymal transition process regulates different aspects of tumorigenesis, but the definition of a unique signaling and regulatory paradigm useful to predict and explain this heterogeneity is still missing. Currently, experimental and computational collected data defined the presence of multiple regulatory networks that are differentially activated at tissue and cellular level, and coordinated by signaling pathways that are activated by multiple stimuli. As demonstrated, this is a tricky problem as the combined action of two or more stimuli, significantly and differentially coordinate the expression of epithelial and mesenchymal markers driving cells into multiple or hybrid transition phases. From the above, it is clear that system biology strategies were applied to address EMT complexity with a significant improvement of our knowledge of the mechanisms related to EMT biology, including the identification of modified pathways, regulatory modules, interaction networks, and EMT biomarkers. These results can be explained taking into account the advanced analytical capabilities of several high-throughput approaches such as mass spectrometry (MS)-based proteomics, and the development of integrative strategies for biological networks identification. This synergy between proteomics and genomics was extremely important to define tumor subtypes with a different EMT signature and new EMT markers by computational analysis. This approach advances MS to complement the genomic characterization of EMT and is likely applicable to other metabolites. In this scenario, the perturbed metabolic network that emerged from different studies should be applicable to the generation of a more global integrated EMT network of regulation.

## Author Contributions

DV conceived and wrote the manuscript. DDS provided critical revisions of the text.

## Conflict of Interest Statement

The authors declare that the research was conducted in the absence of any commercial or financial relationships that could be construed as a potential conflict of interest.
